# Glucosinolate- and Sulforaphane-Enriched Broccoli Extract as a Potential Dual-Action Antioxidant and Elastase Inhibitor

**DOI:** 10.3390/foods15193431

**Published:** 2026-09-25

**Authors:** Chandrika Chaturvedi, H. P. Vasantha Rupasinghe

**Affiliations:** Department of Plant, Food, and Environmental Sciences, Faculty of Agriculture, Dalhousie University, Truro, NS B2N 5E3, Canada

**Keywords:** valorization, *Brassica oleracea*, ultrasonication, extraction, phytochemicals, cosmeceuticals

## Abstract

Broccoli (*Brassica oleracea* var. italica) is a widely cultivated cruciferous vegetable recognized for its rich content of bioactive phytochemicals with documented health-promoting properties. Fresh broccoli florets represent a rich yet underutilized source of bioactive phytochemicals, particularly glucosinolates, with established health benefits. This study optimized ultrasonic-assisted extraction (UAE) using aqueous ethanol to recover glucosinolates and sulforaphane. Total glucosinolate content (TGC) was quantified spectrophotometrically, while sulforaphane, glucoraphanin and glucobrassicin were determined by ultra-performance liquid chromatography–electrospray ionization–mass spectrometry (UPLC-ESI-MS). Under the optimized UAE conditions (25% ethanol, 41 °C, 32 min), the predicted yield of TGC was 46.6 mg of sinigrin equivalents (SE)/g dry weight (DW), while the actual yield achieved was 36.6 ± 4.4 mg of SE/g DW. Among the compared extraction methods, UAE with 25% ethanol yielded the highest TGC, ferric reducing antioxidant power (FRAP), and oxygen radical absorbance capacity (ORAC) with values of 35.5 ± 1.26 mg of SE/g DW, 21.2 ± 2.6 µmol of Trolox equivalents (TE)/g DW, and 156 ± 15.2 µmol of TE/g DW, respectively, with TGC and FRAP being significantly higher than all other methods (*p* < 0.05), while ORAC differences were not statistically significant. In vitro anti-elastase activity was the greatest in the extracts prepared using shaker-assisted extraction (IC_50_ of 0.51 mg/mL) and UAE-25% ethanol (IC_50_ of 0.86 mg/mL). Collectively, these findings position UAE with aqueous ethanol as a promising extraction approach for recovering glucosinolates and sulforaphane from broccoli florets for cosmeceutical applications, providing a basis for future scale-up and process optimization studies.

## 1. Introduction

Broccoli (*Brassica oleracea* var. italica), an economically significant cruciferous vegetable from the Brassicaceae family, is widely recognized for its nutritional profile and abundance of bioactive phytochemicals with health-promoting properties [1,2]. Fresh broccoli florets contain a wide range of bioactive phytochemicals, including glucosinolates, carotenoids, (poly)phenols, and triterpenes, which exhibit significant biological activities, including antimicrobial, antioxidant, anticancer, immunomodulatory, and cardioprotective properties [2]. Among these, glucosinolates, a class of sulfur-containing secondary metabolites, and their hydrolytic products (isothiocyanates) such as sulforaphane have gained significant attention due to their potent role in detoxifying carcinogenic substances [3]. When plant cellular integrity is mechanically disrupted through cutting, chewing or grinding, the endogenous enzyme myrosinase (*β*-thioglucosidase glucohydrolase) comes into contact with substrates, catalyzing the hydrolysis of glucosinolates into biologically active compounds such as nitriles and isothiocyanates, including sulforaphane, indole-3-carbinol, and phenethyl isothiocyanate [4,5,6]. Sulforaphane, in particular, is known to modulate oxidative stress by inducing phase II detoxification enzymes and scavenging reactive oxygen species (ROS), thereby contributing to oxidative homeostasis and anti-aging effects [7].

Skin aging is a complex biological process influenced by both intrinsic and extrinsic factors, with ROS playing a central role in initiating molecular and structural damage [8]. One of the key enzymatic contributors to this process is elastase, a serine protease that catalyzes the degradation of elastin, an essential protein responsible for maintaining the elasticity and firmness of the skin [9]. Overactivation of elastase, whether due to intrinsic aging or external factors such as ultraviolet radiation and ROS, contributes to the breakdown of elastic fibres, leading to skin sagging, wrinkle formation, and dermal thinning, thereby accelerating cutaneous aging [9]. Consequently, the inhibition of elastase activity is considered a promising therapeutic and cosmeceutical target for mitigating age-related skin deterioration. Natural plant-derived compounds with antioxidant and elastase-inhibitory properties are therefore of significant interest in dermatological and cosmetic research [9,10,11].

Efficient extraction of bioactive phytochemicals from plant matrices is essential for their subsequent application as anti-elastase agents of cosmeceuticals. Conventional extraction techniques such as percolation, Soxhlet extraction, shaker-assisted extraction, and hydro-distillation have major drawbacks, including the use of toxic organic solvents, high energy consumption, long extraction times, and high temperatures, which may degrade thermolabile compounds [4]. In contrast, sustainable extraction technologies such as ultrasound-assisted extraction (UAE) using aqueous ethanol have emerged as sustainable alternatives that enhance extraction efficiency and food compatibility, while maintaining phytochemical integrity [5]. UAE facilitates cell disruption and mass transfer through acoustic cavitation, promoting the release of intracellular phytochemicals with minimal thermal degradation [5].

The objective of this research was to optimize UAE-aqueous ethanol for extracting glucosinolates and sulforaphane from broccoli florets. First, optimum conditions of % solvent, temperature, and extraction time were estimated using response surface methodology (RSM) combined with central composite design (CCD). Then, the performance of the optimized method was further compared to the conventional and other green/sustainable extraction methods. In addition, the antioxidant capacity and anti-elastase activity of the resulting extracts were evaluated for their potential protective effects against oxidative stress and enzymatic degradation associated with skin aging.

## 2. Materials and Methods

### 2.1. Chemicals and Reagents

All solvents and reagents were of analytical grade. Diethyl ether, 2,2-diphenyl-1-picrylhydrazyl (DPPH), anhydrous ethanol, methanol, formic acid, gallic acid, *n*-hexane, hydrochloric acid, ferricyanide, monobasic phosphate, dibasic phosphate, sodium acetate trihydrate, glacial acetic acid, 2,4,6-tri(2-pyridyl)-*s*-triazine (TPTZ), ferric chloride, acetonitrile, iron(III) chloride hexahydrate, sodium carbonate, sodium chloride, sodium hydroxide, 6-hydroxy-2,5,7,8-tetramethylchroman-2-carboxylic acid (Trolox), fluorescein sodium salt, 2,2′-azobis(2-methyl-propane) dihydrochloride (AAPH), elastase enzyme, porcine pancreas, N-succinyl-Ala-Ala-Ala-*p*-nitroanilide (AAAPVN), Tris-HCl buffer, choline chloride, citric acid and sinigrin, glucoraphanin, glucobrassicin, sulforaphane, epigallocatechin-3-gallate (EGCG), and quercetin were obtained from Sigma-Aldrich (Oakville, ON, Canada). Deionized water was produced using a Milli-Q purification system (Millipore, Bedford, MA, USA).

### 2.2. Sample Collection

Fresh broccoli heads were purchased from a local market (Sobeys, Bible Hill, NS, Canada) across multiple batches over the course of the experimental period and cut into approximately 2 cm pieces. The broccoli was sourced from a single commercial retailer. As is typical with commercially sourced produce, detailed information on the cultivar, geographical origin, maturity stage, and batch number was not available on the product packaging or from the retailer, and therefore could not be reported. This limitation should be considered, as glucosinolate and sulforaphane contents can vary considerably with these factors. The samples were flash-frozen in liquid nitrogen and stored at −80 °C to preserve their bioactive phytochemicals. The frozen samples were freeze-dried at −40 °C for 72 h using a freeze dryer (Kinetics, FTS Systems Inc., Stone Ridge, NY, USA), followed by grinding with a standard coffee grinder (Model 80393C Hamilton Beach Brands Inc., Glen Allen, VA, USA) and sieving to obtain a uniform particle size of 180 μm. The powdered broccoli was then stored in an airtight container at −20 °C until further extraction experiments.

### 2.3. Experimental Design

This study utilized RSM, a statistical approach widely applied in process optimization, particularly when multiple factors and response variables are involved. A CCD framework was used to evaluate the effects of three independent variables, including ethanol concentration (% *v*/*v*, *X*_1_), extraction temperature (°C, *X*_2_), and extraction time (min, *X*_3_) at five coded levels: low (−1), center (0), high (+1), and two axial points (−α and +α) (Table 1). A total of 20 experimental runs were conducted to evaluate the effects of independent variables on total glucosinolate content (TGC). To ensure data reliability and reproducibility, each of the 20 experimental runs designed by RSM was performed in triplicate. Throughout all experiments, the solid-to-solvent ratio was consistently maintained at 1:20 (*w*/*v*). The data were analyzed using Minitab^®^ statistical software (version 22), and the results are presented in Table 2. Based on the CCD results, optimum conditions were identified and subsequently validated.

### 2.4. Extraction, Purification, and Isolation of Glucosinolates and Sulforaphane Using Ultrasound-Assisted Extraction (UAE) of Broccoli Powder

UAE was conducted using a sonication water bath (ultrasonic frequency of 40 kHz and ultrasonication power of 100 W; model VWR 750D, Avantor, Mississauga, ON, Canada) to extract glucosinolates and sulforaphane from freeze-dried broccoli powder. This technique utilizes high-intensity sound waves to generate cavitation bubbles within the extraction medium. The rapid collapse of these bubbles produces localized shear forces, which effectively disrupt the plant cell walls and enhance the diffusion of bioactive compounds into the solvent. For the extraction process, 0.5 g of freeze-dried broccoli powder was mixed with 10 mL of aqueous ethanol prepared at the required concentration (% *v*/*v*) by diluting anhydrous ethanol with deionized water, at the levels determined by the CCD (Table 2). The samples were vortexed to ensure uniform dispersion before undergoing UAE at different temperatures and extraction times using a vortex (model MSE Pro, Fisher Scientific Inc., Ottawa, ON, Canada). Sonication was carried out in the 40 kHz ultrasonic water bath operated at 100 W (model VWR 750D, Avantor, Mississauga, ON, Canada). The bath temperature was set and monitored throughout to maintain the target extraction temperature specified for each run, and the sample tubes were positioned upright at the centre of the bath and immersed to a consistent depth, with the sample level kept below the surrounding water level to ensure uniform acoustic exposure. After sonication, the extracts were centrifuged at 1957× *g* for 15 min to separate the supernatant from the solid residue using a centrifuge (Sorvall ST 18 centrifuge, Thermo Scientific Inc., Ottawa, ON, Canada) to separate supernatant and pellet formation. The supernatant was then filtered through a 0.20 µm PTFE syringe filter and stored at −20 °C for further analysis. Following extraction, TGC was determined spectrophotometrically, and major glucosinolates (glucoraphanin and glucobrassicin) and isothiocyanates (sulforaphane) were identified and quantified using ultra-high-performance liquid chromatography–electrospray ionization–mass spectrometry (UPLC-ESI-MS).

To comprehensively assess the extraction efficiency of the optimized UAE protocol, a comparative evaluation was performed against other commonly employed extraction techniques reported in the literature. These included microwave-assisted extraction (MAE) (CEM Corporation, Matthews, North Carolina 28106, USA), water bath (Model WS27 Sheldon Manufacturing Inc., Cornelius, IL, USA), shaker-assisted extraction, and natural deep eutectic solvent (NADES)-based extraction (choline chloride:citric acid in a 1:2 molar ratio, consistent with the established literature. The preparation method involved heating at 70 °C with stirring until a homogeneous liquid was formed, and D.I. water was added to reduce the viscosity. Each method utilizes distinct operational principles and solvent systems, which can significantly influence the recovery of target bioactive compounds. The comparison aimed to highlight the relative performance of the optimized UAE method in terms of extraction yield, time efficiency, temperature requirements, and solvent polarity. The specific extraction parameters, such as solvent composition, solid-to-liquid ratio, temperature, duration, and applied energy input associated with each technique, were compiled from previously published studies for direct comparison (Table 3).

### 2.5. Determination of Total Glucosinolate Content (TGC)

Chlorophyll, a major pigment in plant-based extracts, can interfere with the analysis and bioactivity assessment of glucosinolates. To effectively remove chlorophyll while preserving the integrity of glucosinolates, a sequential solvent extraction method was employed. Initially, 1.25 g of broccoli powder was mixed with 222 mL of ethanol, 100 mL of deionized (DI) water, and 28 mL of acetonitrile. This mixture underwent UAE at 20 °C for 20 min. A 100 mL aliquot of *n*-hexane, a nonpolar solvent, was added to the broccoli extract to selectively solubilize chlorophyll due to its hydrophobic nature. This step allows for the separation of chlorophyll from the more polar glucosinolates, which remain in the aqueous phase. The mixture was thoroughly vortexed, followed by centrifugation to facilitate complete phase separation. This biphasic separation enables efficient removal of chlorophyll, ensuring a partially purified glucosinolate and sulforaphane extract suitable for the TGC assay. Chlorophyll separation was conducted on all extracts utilized in the TGC assay for each of the 20 experimental runs.

The TGC was quantified using the previously established method [6,7]. The broccoli extract was hydrolyzed by adding 2 mL of 2 M NaOH to each sample, followed by a 30-min incubation period at room temperature to release 1-thioglucose and isothiocyanate. The reaction was neutralized by adding 310 μL of 1 M HCl and centrifuged at 704× *g* for 5 min. The clarified supernatant was collected, and the reaction was continued by adding 1 mL of 2 mM potassium ferricyanide. Absorbance was measured at 420 nm after 2 min of incubation using a plate reader (Infinite^®^ 200 PRO, TECAN, Mannedorf, Switzerland). The sinigrin standard curve was prepared and serially diluted using freshly made 2 M NaOH as the diluent [6,7]. A 10 mM stock solution of sinigrin was prepared by dissolving 0.394 g of sinigrin in 100 mL of 2 M NaOH. Serial dilutions of the stock solution were prepared to obtain final concentrations of 0.031 to 1 mM, each with a final volume of 2 mL. The solutions were incubated and subjected to the reaction as described above.

A blank of phosphate buffer was used as the reference, and the results were expressed as mg of sinigrin equivalent (SE) per g of dry weight (DW):

$$c=\frac{\Delta OD-intercept}{slope}\;\times \;\frac{Vd}{m}$$

where *c* = concentration of glucosinolates (mmol/kg), Δ*OD* = change in optical density (420 nm) caused by the solution, slope, and intercept values obtained from the sinigrin calibration curve, *V* = volume of extraction solvent used (L), *d* = dilution factor, and *m* = mass of broccoli sample used (kg).

### 2.6. Determination of Glucoraphanin, Glucobrassicin and Sulforaphane Using UPLC-ESI-MS

Quantification of two major glucosinolates (glucoraphanin and glucobrassicin) and isothiocyanate (sulforaphane) of broccoli was performed using the UPLC system with a Waters TQS electrospray ionization (ESI) and quadrupole mass spectrometry (MS) detector (Acquity H-class and Xevo TQ-S, Waters Corporation, Milford, MA, USA), managed with MassLynx v4.2 software. The separation of analytes was performed on a reverse-phase HSS T3 column (100 mm × 2.1 mm i.d., particle diameter of 1.7 μm) (Waters, Milford, MA, USA). Before injection, the extracts were filtered through a 0.2 µm nylon filter and transferred to amber vials (Millipore, Bedford, MA, USA). The chromatographic separation was carried out at a flow rate of 0.4 mL/min with an injection volume of 5 μL. For sulforaphane, the mobile phase consisted of 0.1% formic acid in water (solvent A) and 0.1% formic acid in acetonitrile (solvent B). A linear gradient was used as follows: (time in min, B%): (0, 8%), (1, 8%), (10, 99%), (11, 99%), and (12, 8%). Sulforaphane was analyzed in ESI+ mode with a mass-to-charge ratio (*m*/*z*) of 177.94 (retention time 4.55 min) with a cone voltage of 30 V and a desolvation temperature of 200 °C. For glucoraphanin and glucobrassicin, the same solvents were used in a linear gradient of (time in min, B%): (0, 0%), (3, 0%), (6, 5%), (10, 90%), (11, 90%) and (12, 0%). The detection was carried out in ESI- mode with *m*/*z* of 445.09 for glucobrassicin (retention time 7 min) and *m*/*z* of 434.02 for glucoraphanin (retention time 0.97 min). Commercial standards (purity > 98%) were used to generate calibration curves, and results are expressed in µg/g DW.

### 2.7. Ferric-Reducing Antioxidant Power (FRAP) Assay

The total antioxidant capacity of the glucosinolate and sulforaphane-rich broccoli extract was determined using the FRAP assay [12,13]. The FRAP reagent was prepared by combining three freshly prepared solutions: (i.) 300 mM acetate buffer (pH 3.6), prepared by dissolving 3.1 g of sodium acetate trihydrate and adding 16 mL of glacial acetic acid in 1 L of deionized water; (ii.) 10 mM of 2,4,6-tri(2-pyridyl)-*s*-triazine (TPTZ) solution, prepared by dissolving 150 mg of TPTZ in 50 mL of 40 mM HCl; and (iii.) 20 mM of ferric chloride solution, prepared by dissolving 270 mg of FeCl_3_·6H_2_O in 50 mL of distilled water. The working FRAP reagent was prepared by combining these three solutions in a 10:1:1 ratio (acetate buffer:TPTZ:FeCl_3_) and preheated to 37 °C before use. For FRAP analysis, 10 µL of broccoli extract was mixed with 300 µL of working FRAP reagent in a 96-well plate (COSTAR 9017, Fisher Scientific, Ottawa, ON, Canada) and incubated for 5 min at 35 °C in the dark to allow full chromophore development. The final-colored sample absorbance was measured at 593 nm using a Tecan plate reader (Infinite^®^ 200 PRO, TECAN, Mannedorf, Switzerland) with blank subtraction and a calibration curve using Trolox standards (5–450 µM, R^2^ ≥ 0.995). FRAP results were expressed as µmol Trolox equivalents per g DW (µmol TE/g DW), with triplicate measurements.

### 2.8. 2,2-Diphenyl-1-picrylhydrazyl (DPPH) Radical Scavenging Assay

The antioxidant activity was also assessed using the DPPH radical scavenging assay, adapted for high-throughput analysis in 96-well microplates [4]. Broccoli extracts were prepared at concentrations ranging from 0.5 to 40 mg/mL (expressed on a dry extract mass basis) and mixed in equal volumes (150 µL each) with a freshly prepared 0.2 mM DPPH solution (0.0079 g in 100 mL methanol). The reaction mixtures were incubated in the dark at 25 °C for 30 min to prevent photodegradation of the radical. Absorbance was measured at 517 nm using a plate reader (Infinite^®^ 200 PRO, TECAN, Mannedorf, Switzerland). Antioxidant activity was determined based on the IC_50_ value, defined as the concentration of extract required to inhibit 50% of the initial DPPH radical.

### 2.9. Oxygen Radical Absorbance Capacity (ORAC) Assay

The ORAC assay was performed using a previously established protocol [14]. The samples, Trolox standards, fluorescein sodium salt (0.957 μM), and AAPH (150 mM) were prepared in 75 mM phosphate buffer (pH 7.0). The reaction was conducted in black 96-well microplates, where 35 μL of each sample or Trolox standard was mixed with 130 μL of fluorescein salt. The plate was pre-incubated in a plate reader at 37 °C for 30 min. After incubation, 35 μL of pre-warmed (37 °C) AAPH solution was added to the appropriate wells to initiate the reaction, bringing the total volume to 200 μL. The assay was carried out at 37 °C for approximately 20 min, with fluorescence readings taken every 60 s at an excitation wavelength of 490 nm and an emission wavelength of 540 nm. The antioxidant activity was determined by calculating the area under the fluorescence decay curve (AUC) for standards made from 2 to 150 µM concentrations. The results were expressed as μmol Trolox equivalents per g DW (μmol TE/g DW).

### 2.10. Anti-Elastase Activity Assay

The elastase inhibition assay was performed following a modified protocol from Millipore Sigma (MilliporeSigma Canada Ltd. Oakville, ON, Canada) with some modifications. The elastase inhibitory activity of the tested extracts was assessed using porcine pancreatic elastase (0.3 U/mL) prepared in a cold 100 mM Tris-HCl buffer (pH 8.0 at 25 °C). The extracts were pre-incubated with the enzyme for 15 min at room temperature in the dark. The substrate, N-succinyl-Ala-Ala-Ala-*p*-nitroanilide (AAAPVN), was prepared at 4.4 mM in Tris-HCl buffer. The final reaction mixture (250 μL) contained 96.7 mM of Tris-HCl, 0.29 mM of AAAPVN, and 0.02 U of elastase. The reaction was initiated by substrate addition, and absorbance was measured at 410 nm continuously for 20 min using a plate reader (Infinite^®^ 200 PRO, TECAN, Mannedorf, Switzerland). Epigallocatechin gallate (EGCG) (250 μM, 0.114 mg/mL) and distilled water were used as positive and negative controls, respectively. To determine IC_50_ value, test extracts were evaluated at multiple concentrations (0.1–15 mg/mL).

### 2.11. Statistical Analysis

Data were expressed as mean ± SEM. Statistical analyses were conducted using Minitab version 22.5 (Minitab LLC, State College, PA, USA). For glucosinolate isolation optimization, response surface methodology (RSM) combined with regression models and one-way ANOVA determined the optimal conditions. For data analysis, a general linear model followed by Tukey’s post hoc tests for group comparisons was employed. Statistical significance was set at *p* < 0.05. All triplicate measurements represent independent extraction replicates rather than technical replicates of a single extract. Total glucosinolate content (TGC) was used as the primary criterion for optimizing the extraction conditions; sulforaphane yield was not optimized independently but was evaluated under the TGC-optimal conditions as a secondary outcome. The separate optima observed for each response reflect that each response surface was optimized individually.

## 3. Results

### 3.1. Optimization of the Extraction of Glucosinolates and Sulforaphane from Broccoli Florets

**Figure 1:**
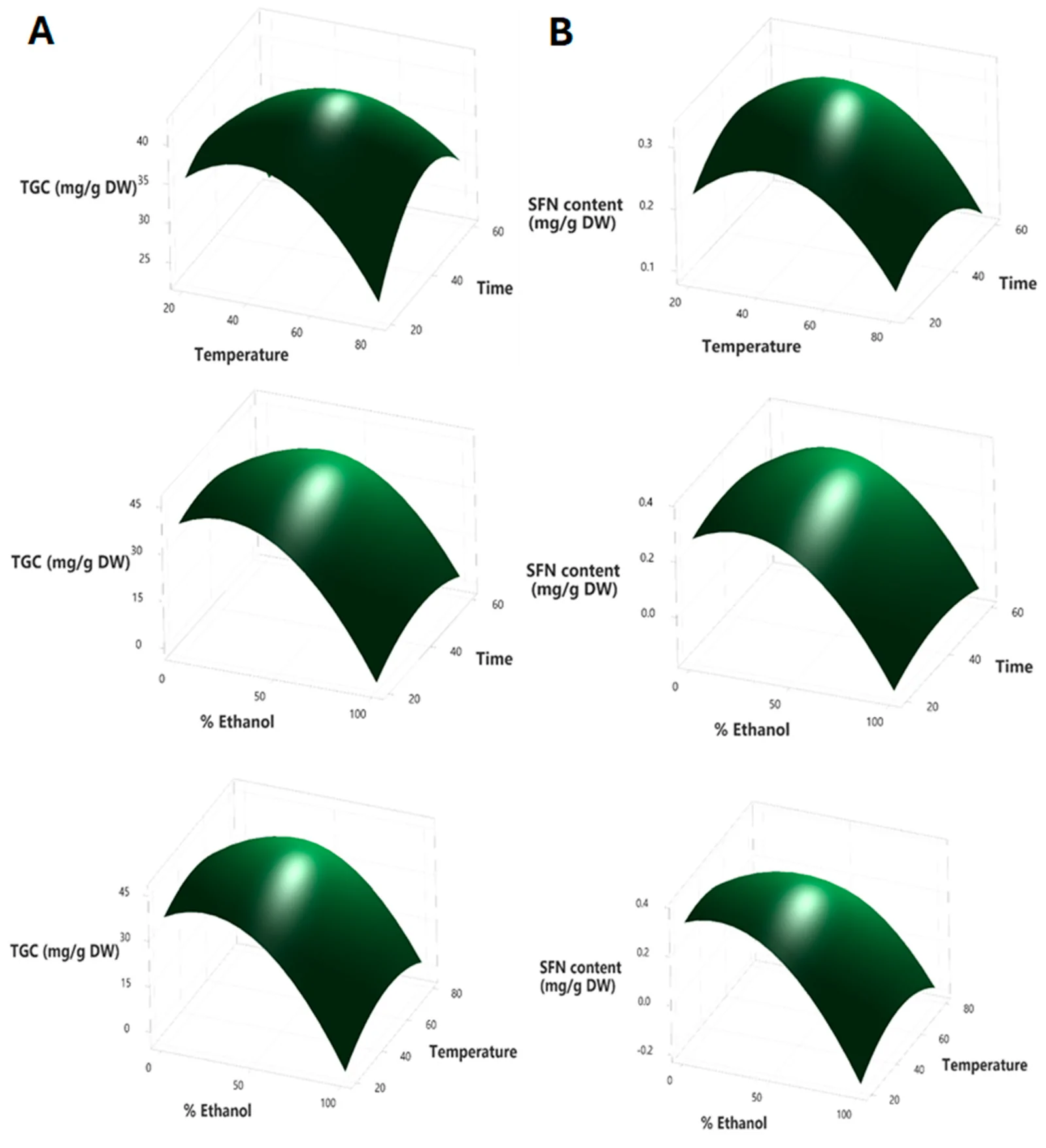
Three-dimensional response surface plots generated by the model depict how variations in solvent concentration, extraction temperature, and time influence these responses. (**A**) Total glucosinolate content (TGC, mg SE/g DW). (**B**) Sulforaphane content (SFN, mg/g DW) as functions of ethanol concentration, extraction temperature, and time. Plots show pairwise interactions: temperature vs. time, EtOH (%) vs. time, and EtOH (%) vs. temperature. SE, sinigrin equivalence; DW, dry weight; EtOH, ethanol.

A three-factor CCD was employed to evaluate the influence of independent variables on the total glucosinolate and sulforaphane content using a spectrophotometric method and UPLC-ESI-MS analysis, respectively (Table 2). A total of 20 experimental runs were conducted using varying extraction conditions involving ethanol concentration (*X*_1_: 0–100%), temperature (*X*_2_: 20–80 °C), and extraction time (*X*_3_: 20–60 min). TGC ranged from 11.0 to 46.8 mg SE/g DW, with the maximum yield obtained under optimum extraction conditions of 25% ethanol, 41 °C, and 32 min. Sulforaphane content varied from 0.01 to 0.36 mg/g DW, peaking at 23.2% ethanol, 36 °C, and 38 min, closely aligning with the optimum conditions for recovering TGC. The 3D response surface plots illustrate the influence of interaction effects between independent variables on the model responses (Figure 1).

**Figure 2:**
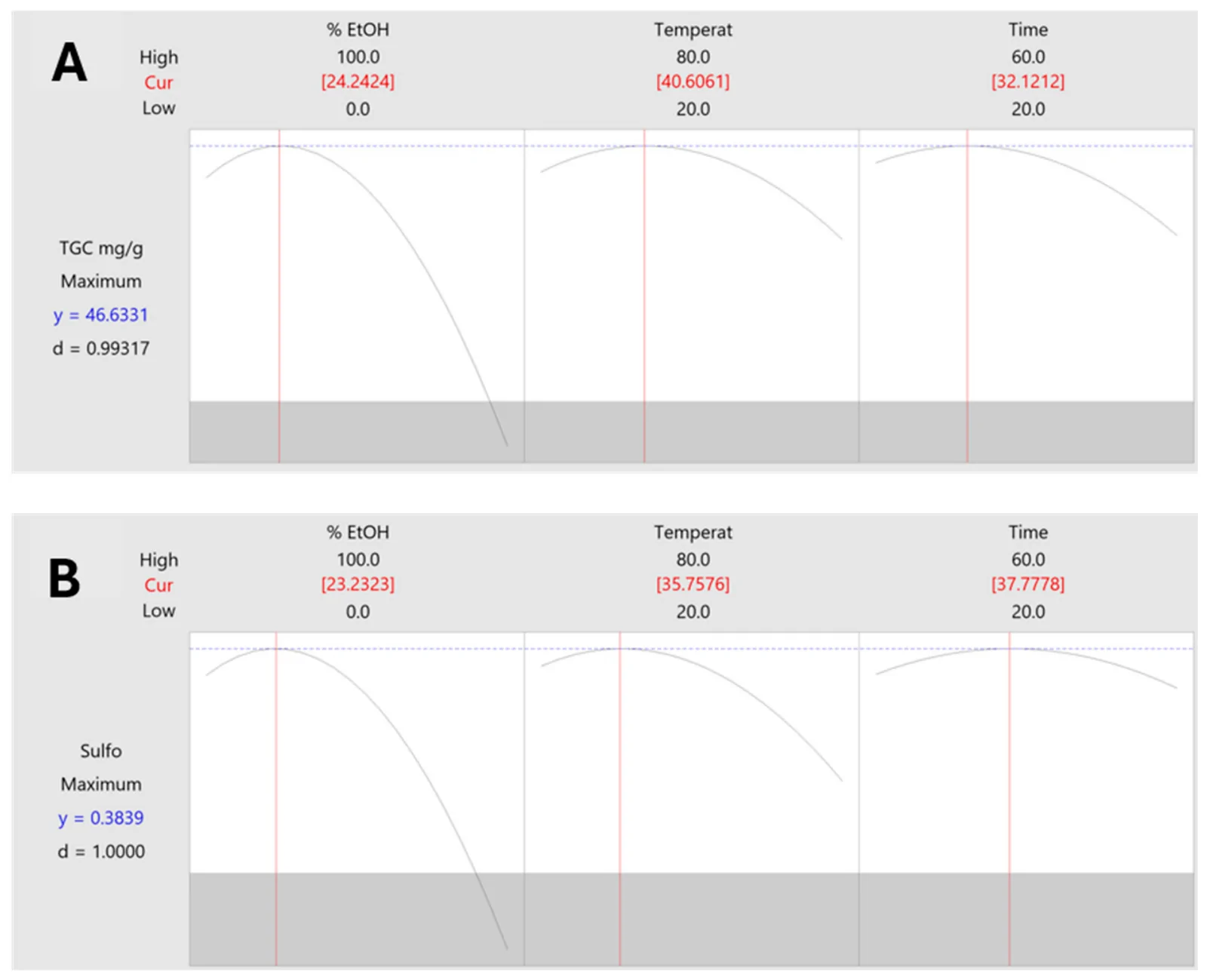
The optimization plots illustrate the effect of extraction parameters on two key response variables: (**A**) total glucosinolate content (TGC) (expressed as mg sinigrin equivalents per g DW) and (**B**) sulforaphane content (mg/g DW). %EtOH, ethanol; temperat, temperature; sulfo, sulforaphane; DW, dry weight.

The 3D response surface plots illustrate the interactive and nonlinear effects of ethanol concentration, extraction temperature, and extraction time on TGC and sulforaphane content. TGC increases with low ethanol concentrations and extraction temperature but declines at higher levels, indicating a quadratic relationship among these variables. At a fixed ethanol concentration, increasing the extraction temperature initially enhanced TGC. However, at higher extraction temperatures, TGC decreased, as indicated by the downward curvature in the surface plots. Similarly, extraction time had a significant effect, where longer extraction time beyond the optimum conditions (32 min) reduced TGC (Figure 2).

### 3.2. UAE Model Fitting

To assess the influence of independent variables on the TGC and sulforaphane content, a second-order polynomial regression model was validated through analysis of variance (ANOVA). The ANOVA results (Table 4) revealed significant variation (*p* ≤ 0.05) across different combinations of ethanol concentration, extraction temperature, and extraction time. Ethanol concentration (*X*_1_) exerted a highly significant effect (*p* < 0.05) on both TGC and sulforaphane content. In contrast, extraction temperature (*X*_2_) and time (*X*_3_) showed no significant effect on TGC (*p* > 0.05), although temperature displayed a marginal influence on UPLC-ESI-MS response (*p* = 0.057). Quadratic effects of ethanol concentration and temperature were statistically significant (*p* < 0.05) for both responses, whereas the quadratic effect of time was significant only for TGC (*p* < 0.05) and non-significant for UPLC-ESI-MS (*p* > 0.05). All interaction terms (*X*_1_*X*_2_, *X*_1_*X*_3_, and *X*_2_*X*_3_) were non-significant (*p* > 0.05), indicating minimal synergistic effects among the variables. Despite a significant lack-of-fit (*p* < 0.05), the models showed strong predictive power with R^2^ values of 93.97% (TGC) and 89.13% (sulforaphane), and adjusted R^2^ of 88.54% and 79.35%, respectively. The complete ANOVA table with all statistical parameters, together with residual diagnostic plots (normal probability plots of the residuals and plots of residuals versus fitted values), is provided as Supplementary Material (Figure S1). These diagnostics were examined to assess model adequacy: the residuals were approximately normally distributed and randomly scattered about zero, indicating no major violation of model assumptions. The significant lack-of-fit is attributable largely to the very small pure-error variance among the closely agreeing replicated centre points; given the high R^2^ and adjusted R^2^ values and the acceptable residual behavior, the models were considered adequate for prediction within the experimental range.

The generated equation computed for the polynomial model is given below in coded variables:

TGC (mg SE/g DW) = 26.7 + 0.147 (*X*_1_) + 0.388 (*X*_2_) + 0.635 (*X*_3_) − 0.00737 (*X*_1_ *X*_1_) − 0.00846 (*X*_2_*X*_2_) − 0.01604 (*X*_3_*X*_3_) + 0.00240 (*X*_1_*X*_2_) + 0.00368 (*X*_1_*X*_3_) + 0.00754 (*X*_2_*X*_3_)

Sulforaphane (mg/g DW) = 0.038 + 0.00156 (*X*_1_) + 0.00716 (*X*_2_) + 0.0104 (*X*_3_) − 0.000083 (*X*_1_ *X*_1_) − 0.000110 (*X*_2_*X*_2_) − 0.000134 (*X*_3_*X*_3_) + 0.000047 (*X*_1_*X*_2_) + 0.000014 (*X*_1_*X*_3_) − 0.000012 (*X*_2_*X*_3_)

### 3.3. Model Validation and Comparison of Predicted vs. Actual Yields

To validate the predictive strength of the RSM-derived quadratic model, experimental runs were conducted under optimized UAE conditions (25% ethanol, 41 °C, and 32 min), and both TGC and sulforaphane content were determined and compared with the predicted values (Table 5).

### 3.4. Glucosinolate Profiles of Broccoli Extracts Using UPLC-ESI-MS Analysis

Seven extraction techniques, including conventional (e.g., shaker and water bath) and green/sustainable extraction methods (e.g., UAE, MAE, and NADES), were evaluated for glucosinolates and sulforaphane recovery from broccoli. The resulting extracts were analyzed for TGC, total antioxidant capacity (FRAP, DPPH, and ORAC), and elastase inhibition activity to evaluate their bio-functional potential.

**Figure 3:**
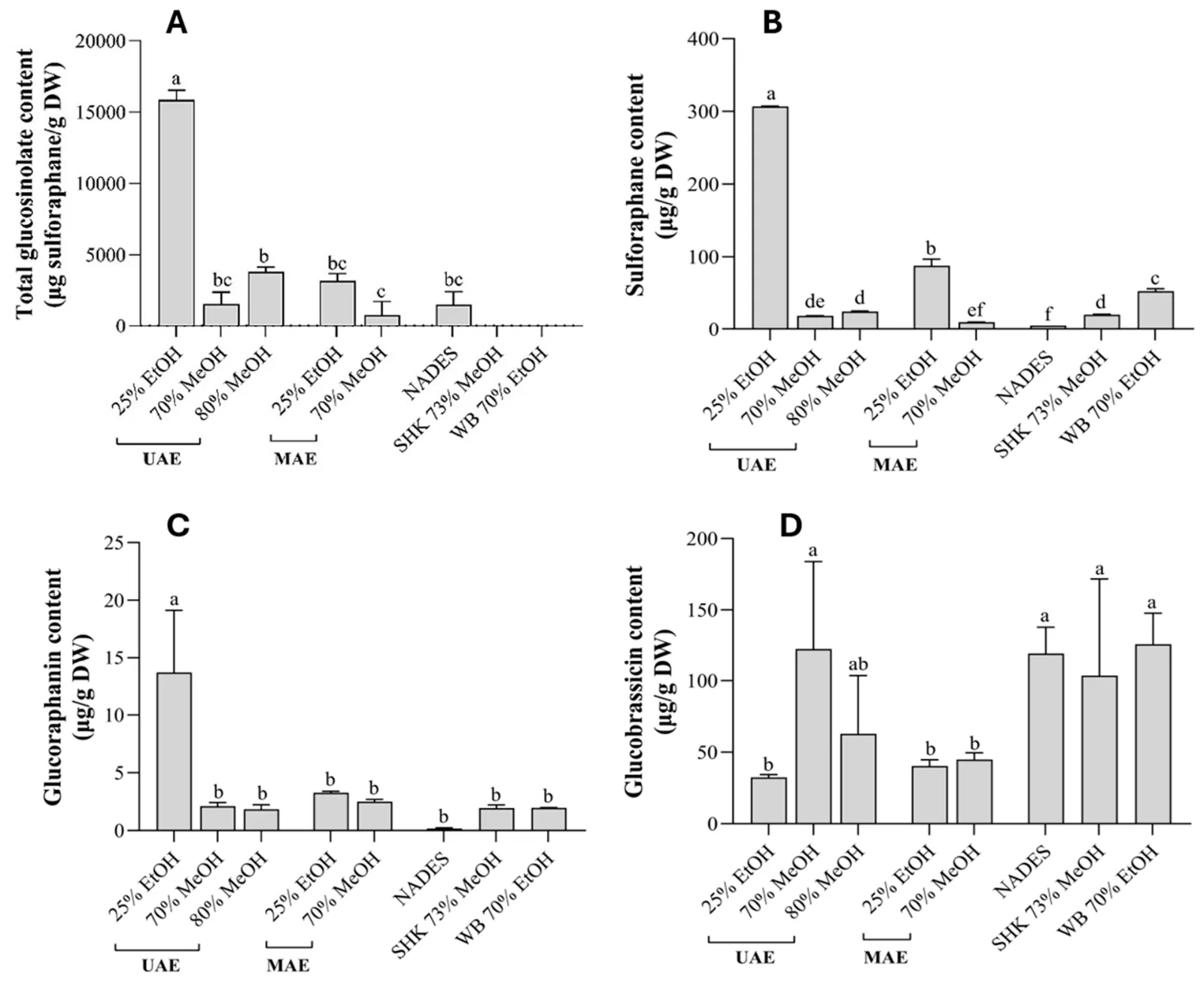
The total glucosinolate content (TGC) (**A**), sulforaphane content (**B**), glucoraphanin content (**C**) and glucobrassicin content (**D**) in relation to the different extraction processes. Abbreviations: UAE, ultrasound-assisted extraction; WB, water bath; MAE, microwave-assisted extraction; NADES, natural deep eutectic solvent; DW, dry weight basis; EtOH, ethanol; MeOH, methanol; SHK, shaking water bath. The different letters of means (a–f) indicate significant differences (*p* < 0.05) among them using Tukey’s HSD test.

UAE with 25% ethanol at 41 °C for 32 min exhibited the highest TGC (35.5 ± 1.26 mg SE/g DW), representing 4- to 10-fold higher (*p* < 0.05; Tukey’s HSD) than methanol-based UAE (Figure 3A). Higher polarity solvents (80% and 70% methanol) significantly reduced TGC yields (8.54 ± 0.64 and 3.47 ± 1.55 mg SE/g DW, respectively). MAE with 25% ethanol under identical conditions as UAE-25% ethanol resulted in a significantly lower yield of TGC (7.12 ± 0.90 mg SE/g DW), while MAE at 100 °C with 70% methanol resulted in a drastic decline in TGC (1.75 ± 1.74 mg SE/g DW). The NADES system (choline chloride: citric acid, 1:2 molar ratio) at 70 °C for 1 h generated comparable yields (3.43 ± 1.59 mg SE/g DW) to MAE with 70% methanol. Conventional extraction (shaker and water bath) failed to extract detectable sulforaphane, indicating the necessity of energy-assisted techniques.

UPLC-ESI-MS analyses revealed that the concentrations of key organosulfur compounds glucoraphanin, glucobrassicin, and sulforaphane varied significantly depending on the extraction technique employed (Figure 3B–D). Among all extraction methods, UAE with 25% ethanol at 41 °C for 32 min demonstrated the highest sulforaphane yield (307 ± 0.83 µg/g DW), which was statistically higher (*p* < 0.05) than other tested extraction techniques. Notably, UAE-25% ethanol also produced the highest recovery of glucoraphanin (13.6 ± 5.47 µg/g DW), significantly outperforming the remaining extraction methods (*p* < 0.05). Glucobrassicin content varied significantly across extraction conditions, ranging from 32.5 to 126 µg/g DW. Comparable, elevated glucobrassicin levels were observed using water-bath extraction (126 ± 21.8 µg/g DW), UAE with 70% methanol (123 ± 61.4 µg/g DW), NADES (120 ± 18.5 µg/g DW), and shaker extraction (104 ± 68.1 µg/g DW), with no statistically significant differences observed among these methods (*p* > 0.05), indicating comparable extraction efficiencies for glucobrassicin. In contrast, both UAE and MAE with 25% ethanol or 70% methanol produced significantly lower yields of 32.5 to 44.9 µg/g DW. The TGC data were consistent with UPLC-ESI-MS quantification of sulforaphane, with UAE with 25% ethanol yielding the highest sulforaphane content.

### 3.5. Total Antioxidant Capacity

The antioxidant capacity of broccoli extracts obtained from various extraction methods was assessed using FRAP, DPPH, and ORAC assays (Table 6). FRAP values varied significantly across extraction methods, ranging from 12.8 ± 0.06 to 21.1 ± 2.5 µmol TE/g DW. UAE-25% ethanol exhibited the highest reducing capacity (21.1 ± 2.5 µmol TE/g DW), consistent with its greatest sulforaphane and glucosinolate content, whereas MAE with 25% ethanol produced the lowest value (12.8 ± 0.06 µmol TE/g DW). DPPH radical scavenging activity varied among extracts, with MAE 25% ethanol showing the weakest DPPH activity (IC_50_ = 7.06 ± 0.16 mg/mL), consistent with its low FRAP value and moderate TGC, suggesting a relatively diminished antioxidant profile. Conversely, MAE with 70% methanol demonstrated the strongest DPPH radical scavenging activity (IC_50_ = 2.39 ± 0.33 mg/mL). ORAC values across all extracts were comparable (129–156 µmol TE/g DW), with UAE 25% ethanol showing the numerically highest ORAC value, although differences among extracts were not statistically significant (*p* > 0.05), (156 ± 15.2 µmol TE/g DW), and NADES extract showing a similar trend (153 ± 95.4 µmol TE/g DW) (Table 6). Despite the lack of statistical significance, these results align with the FRAP trends, indicating consistent antioxidant potential across the most effective extraction methods.

### 3.6. Anti-Elastase Activity

**Figure 4:**
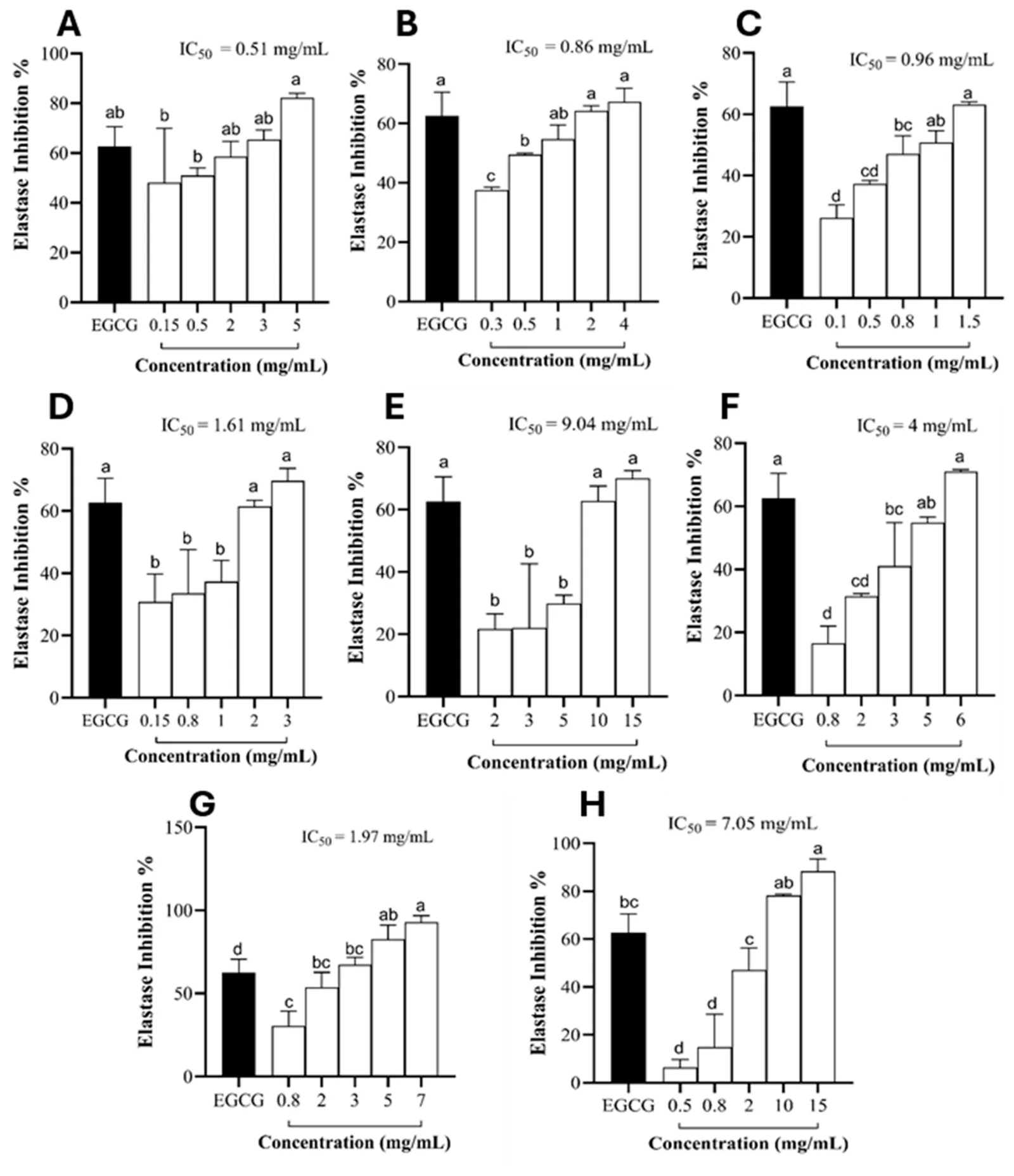
Elastase inhibition (%) of broccoli extracts obtained by different extraction methods and solvent conditions: (**A**) shaker with 73% MeOH, (**B**) UAE with 25% ethanol, (**C**) sulforaphane (reference compound), (**D**) UAE with 80% MeOH, (**E**) MAE with 70% MeOH, (**F**) water bath with 70% EtOH, (**G**) UAE with 70% MeOH, and (**H**) MAE with 25% EtOH. IC_50_ values represent the triplicate. The different letters of means (a–d) indicate the significant differences (*p* < 0.05) among them using Tukey’s HSD test.

The anti-elastase activity of broccoli extracts varied significantly among the extraction methods tested, as expressed by their respective IC_50_ values (Figure 4). Shaker extraction using 73% methanol at 25 °C for 1 h demonstrated the numerically greatest inhibitory potency (IC_50_ = 0.51 mg/mL DW), followed closely by UAE with 25% ethanol (IC_50_ = 0.86 mg/mL DW). Tukey’s post hoc analysis (*p* > 0.05) indicated no significant difference between these two extraction methods, indicating comparable elastase-inhibitory efficacy. For comparison, the pure reference compound, sulforaphane exhibited an IC_50_ of 0.96 mg/mL DW, placing its inhibitory capacity within the range of the most effective extract-based treatments. Moderate inhibition was observed for UAE with 70% and 80% methanol (IC_50_ = 1.97 and 1.61 mg/mL DW, respectively). In contrast, MAE at high temperature (70% methanol, 100 °C, 5 min) exhibited the lowest inhibitory potency (IC_50_ = 9.04 mg/mL DW), followed by MAE with 25% ethanol (IC_50_ = 7.05 mg/mL).

## 4. Discussion

Glucosinolates and their hydrolysis product sulforaphane have attracted considerable scientific attention due to their documented chemoprotective, anti-inflammatory, and dermatological bioactivities, positioning them as valuable constituents in functional foods, nutraceuticals, and cosmeceutical formulations [15,16]. Conventional extraction methods often rely on toxic organic solvents such as methanol or acetonitrile, which pose challenges related to toxicity, environmental impact, and regulatory acceptance [17]. Consequently, the development of sustainable extraction strategies utilizing food-grade solvents has become a priority, particularly approaches based on aqueous ethanol systems that offer improved safety and sustainability profiles [18].

In the present study, UAE using 25% ethanol exhibited the highest TGC (36.6 mg SE/g DW), outperforming both higher ethanol- and methanol-based extractions. The superior performance at this low ethanol concentration (≤50%) can be attributed to the intermediate polarity of aqueous ethanol solutions (polarity index ~7.0), which enhances solvation of both the hydrophilic sulfate and glucose-containing moieties of glucosinolates, facilitating solubilization of aliphatic compounds such as glucoraphanin while limiting the co-extraction of polymeric phenolics and sugars that could affect downstream bioactivity [19,20]. In contrast, extraction with anhydrous ethanol (100%) resulted in substantially lower TGC (11 mg SE/g DW), consistent with the inherently poor solubility of glucosinolates in non-aqueous systems and confirming their hydrophilic nature [21].

Beyond solvent composition, extraction temperature exerted a multifaceted role in glucosinolate recovery. To systematically evaluate the influence of extraction parameters and their interactions on glucosinolate recovery, an RSM approach based on a CCD was employed. Among the tested variables, extraction solvent composition showed the most significant linear effect (*p* < 0.05), whereas extraction temperature (*X*_2_; *p* = 0.525) and extraction time (*X*_3_; *p* = 0.308) were not significant linear factors. For sulforaphane, ethanol concentration was also statistically significant as a linear factor (*p* < 0.05), while temperature showed a marginal effect (*p* = 0.057) and extraction time was not significant (*p* = 0.866). The quadratic effect of extraction time was significant for TGC (*p* = 0.037) but not for sulforaphane (*p* = 0.256). At moderately high temperatures (~41 °C), reduced solvent viscosity, enhanced cell permeability, and accelerated mass transfer resulted in higher extraction of glucosinolates [22,23]. However, excessive heating (>50–60 °C) likely led to thermal degradation and isomerization of labile aliphatic glucosinolates, together with partial inactivation of endogenous myrosinase [24]. At these temperatures, specifier proteins (such as epithiospecifier protein, ESP) may also be denatured, altering the hydrolysis pathway and further influencing the yield of sulforaphane [25]. A previous RSM-based study has reported that mild conditions (25 °C, 2.5–5 min) can achieve comparable glucosinolate recoveries when solvent polarity is optimized [26].

Compared with earlier studies [24,27,28], the optimized UAE-ethanol protocol demonstrates a more sustainable and effective approach for the recovery of glucosinolate and sulforaphane from broccoli florets. A comparative study using rapeseed demonstrated that 80% ethanol extracted only 14% of total glucosinolates, whereas water alone achieved 94% recovery, highlighting the critical role of solvent polarity in glucosinolate solubilization [29]. The poor performance of samples with high ethanol content is also consistent with the physicochemical behavior of aqueous ultrasound systems, i.e., prolonged cavitation in low-polarity solvents can generate localized hotspots and free radicals that accelerate thermal and oxidative degradation of glucosinolates into inactive breakdown products [20,30]. Given that glucosinolates are inherently water-soluble yet thermolabile compounds, they are sensitive to elevated temperatures, pH shifts, and oxidative stress [22,31]. The present results confirm this sensitivity, where extraction times exceeding 32 min resulted in significant reductions in TGC, indicating that extended ultrasound exposure becomes detrimental once the extraction-degradation threshold is surpassed. Similar trends have been reported in broccoli subjected to high-pressure processing, where glucosinolates were largely preserved at 20 °C (100–500 MPa, 15 min) due to limited myrosinase activity, but underwent substantial degradation (>60%) at 40 °C [32]. A similar pattern was reported in an RSM-based UAE study on cauliflower, in which food-grade ethanol (42%), mild temperature (43 °C) and short duration (30 min) produced 7.4 mg of SE/g DW, further emphasizing the importance of balancing solvent polarity, time, and temperature [20]. Although MAE can rapidly disrupt plant tissues, its comparatively lower glucosinolate recovery is likely due to non-uniform dielectric heating and localized thermal hotspots that compromise the stability of thermolabile glucosinolates [8]. Likewise, natural deep eutectic solvents (NADES), despite their strong hydrogen-bonding capacity, have demonstrated moderate extraction efficiencies, primarily due to high viscosity, which imposes significant mass transfer resistance, slowing diffusion kinetics and limiting the release of intercellular glucosinolates [33]. Additionally, the low water activity and strong solvation cages around NADES restrict myrosinase enzyme activity and hinder subsequent sulforaphane formation, further reducing TGC yield.

The UPLC-ESI-MS analysis confirmed the efficient extraction of key glucosinolates and their derivatives, including sulforaphane, glucoraphanin, and glucobrassicin. The chromatograms revealed distinct peaks corresponding to each compound, with retention times of 0.97 min (glucoraphanin), 4.55 min (sulforaphane), and 7.0 min (glucobrassicin), demonstrating effective separation and minimal co-elution. These retention times align with previously reported values for broccoli extracts, where glucoraphanin, sulforaphane, and glucobrassicin typically eluted at 1.35 min, 4.51–6.45 min, and 6.10 min, respectively, supporting the reliability, reproducibility, and analytical accuracy [20,34]. Quantitatively, the sulforaphane concentration determined in the present study was 0.38 mg/g DW, which is comparable to previously reported sulforaphane levels in mature broccoli. Nakagawa and colleagues [35] reported sulforaphane concentrations ranging from 0.69–1.71 mg/g DW in broccoli from Japan, whereas Campas-Baypoli and colleagues [36] reported lower concentrations (0.31–0.45 mg/g DW) in lyophilized broccoli florets from Mexico. The sulforaphane yield obtained in this study falls within the lower range of reported values, suggesting comparable retention and extraction efficiency. Variability among studies is likely attributable to differences in cultivar, agronomic conditions, post-harvest processing, glucoraphanin-to-sulforaphane conversion efficiency, and analytical methodology.

Although glucosinolates are not direct radical scavengers, their enzymatic or chemical hydrolysis yields isothiocyanates such as sulforaphane, which are well recognized for activating the nuclear factor erythroid 2-related factor 2/antioxidant response element (Nrf2/ARE) signaling pathway and phase II detoxification enzymes [37]. This indirect mechanism distinguishes them from classical phenolic antioxidants and highlights the relevance of both chemical antioxidant assays and bioactive precursor content. In the present study, the extract obtained using UAE with 25% ethanol exhibited the highest FRAP value, and although the ORAC differences among extracts were not statistically significant (Table 6), FRAP values corresponded closely to its highest TGC and sulforaphane content (Figure 3). Because the phenolic and other co-extracted constituents of the extracts were not fully characterized, this co-occurrence is correlative only and does not establish that glucosinolate-derived metabolites are directly responsible for the observed reducing capacity and peroxyl-radical quenching. Interestingly, broccoli extracts generated using high-polarity solvents (70–80% methanol) demonstrated comparatively lower FRAP values but stronger DPPH radical scavenging activity. This divergence likely reflects solvent-dependent shifts in phytochemical composition: highly polar solvents enrich phenolics and flavonoid glycosides that efficiently quench stable radicals (DPPH), while extracts of moderately polar solvents contain a broader array of redox-active compounds contributing to FRAP. Given their weak direct radical-scavenging activity, glucosinolates likely play a complementary antioxidant role to phenolics [20,33]. As sulforaphane lacks multiple phenolic hydroxyl groups, it does not efficiently donate hydrogen atoms to stabilize radicals such as DPPH. However, its electrophilic nature may enhance ORAC values due to indirect modulation of intracellular antioxidant pathways and its capacity to neutralize reactive species through conjugation with thiol.

The anti-elastase potential of broccoli extracts was evaluated using porcine pancreatic elastase and expressed as IC_50_ values [38]. Among the tested broccoli extracts, the shaker-assisted extract showed the numerically greatest potency (IC_50_ = 0.51 mg/mL, % inhibition = 90.8%), followed by UAE-25% ethanol (IC_50_ = 0.86 mg/mL, % inhibition = 62.6%). Both extracts outperformed the reference compound sulforaphane (IC_50_ of 0.96 mg/mL DW, % inhibition = 63.2%), suggesting that the synergistic activity of glucosinolate with other phytochemical constituents of the extract enhances elastase inhibition. In contrast, MAE with 70% methanol and 25% ethanol produced moderate inhibition (IC_50_: 9.04 and 7.05 mg/mL, respectively), which aligns with evidence that methanol-based extractions capture a broad metabolite profile, including polyphenols, but also introduce thermal or oxidative degradation during high-energy extraction, possibly reducing inhibitory potency [39]. Comparative analysis with other Brassicaceae extracts further contextualizes these findings. Recent work on *Nasturtium officinale* microshoot (watercress) extracts reported elastase inhibition values of 79.8% for optimized aqueous extracts, exceeding both parent herb material and younger microshoot cultures [40]. Similarly, root extracts of *Eutrema japonicum*, another member of the Brassicaceae family, exhibited exceptionally high inhibition (90.2%), comparable to the shaker-assisted broccoli extract [41]. Although direct elastase inhibition by broccoli extracts has not previously been reported, these results are consistent with their broader anti-aging, anti-inflammatory, and antioxidant activities. Broccoli sprout extract (BSE) has been shown to suppress particulate-matter-induced skin aging by inhibiting p38 mitogen-activated protein kinase (p38 MAPK) signaling cascade and reducing downstream mediators of dermal degeneration, including matrix metalloproteinase-1 (MMP-1), cyclooxygenase-2 (COX-2), and interleukin-6 (IL-6) in human HaCaT keratinocytes [42]. In that study, p38 MAPK activation was reported to be associated with elastase release and extracellular matrix degradation, and BSE was shown to bind p38α MAPK and block its downstream targets. However, these signaling pathways (p38 MAPK, MMP-1, COX-2, and IL-6) were investigated in other studies and were not examined in the present work, which assessed only in vitro elastase inhibition and antioxidant activity; any mechanistic parallels therefore remain speculative. Similarly, broccoli flower extract (BFE) suppresses ultraviolet-B (UVB)-induced MMP-1 expression in primary fibroblasts [43]. Because elastase and MMP-1 both contribute to the degradation of collagen and elastin fibers, these literature findings on MMP-1 are cited for context only; MMP-1 and the associated signaling pathways were not measured in the present study, which evaluated in vitro elastase inhibition. Collectively, these findings highlight the critical influence of solvent type, extraction parameters, and matrix composition in determining the bioactivity of plant-derived elastase inhibitors.

## 5. Conclusions

This study provides the first comprehensive evaluation of glucosinolate- and sulforaphane-rich broccoli extracts obtained through both conventional and sustainable extraction methods, assessing antioxidant capacity and elastase inhibition activity. The RSM identified UAE with 25% ethanol at 41 °C for 32 min as the optimal condition for maximizing the recovery of glucosinolates and their hydrolyzed compound sulforaphane, as validated by spectrophotometric assays and UPLC-ESI-MS analysis. The UAE-25% ethanol extract also exhibited strong in vitro antioxidant capacity (FRAP, DPPH and ORAC) and potent in vitro anti-elastase activity, potentially reflecting complementary contributions from glucosinolates and co-extracted phenolic compounds; however, phenolic content was not directly quantified in this study, and this interpretation remains speculative. Compared to other reported extraction methods, UAE-25% ethanol provided the highest TGC and sulforaphane simultaneously. Collectively, these results support UAE with aqueous ethanol as a sustainable and resource-efficient extraction technique for recovering glucosinolates and sulforaphane from broccoli florets, with potential applications in the development of natural health products, nutraceuticals, and cosmeceuticals. However, scale-up feasibility remains to be established, and future work should evaluate process metrics such as energy consumption, solvent recovery, and throughput under pilot- or industrial-scale conditions.

However, it should be emphasized that these findings are based on in vitro assays; the extract demonstrated inhibition of porcine pancreatic elastase and antioxidant activity, but anti-aging efficacy in human skin was not established. Key limitations include the absence of human skin elastase testing, cell-based assays, dermal penetration studies, and in vivo validation. In addition, the ethanol concentration range (0–100%) would be expected to differentially affect myrosinase activity; therefore, future studies should decouple extraction from enzymatic conversion by controlling pre-hydrolysis conditions, as myrosinase activity was not independently measured in the present study. Future studies should focus on process scale-up, stability of the extract and shelf life, and in vivo validation of anti-elastase activity to enable commercial utilization.

## Figures and Tables

**Table 1:** Experimental range of coded and actual values for central composite design.

Independent Variable	Levels (Ultrasound-Assisted Extraction)				
	−1.68	−1	0	1	+1.68
*X*_1_ Ethanol concentrations (%)	0	20.27	50	79.73	100
*X*_2_ Extraction temperature (°C)	20	32.16	50	67.83	80
*X*_3_ Extraction time (min)	20	28.10	40	51.89	60

**Table 2:** Central composite design and its response variables for total glucosinolate content and sulforaphane of the extracts of freeze-dried broccoli powder.

	Independent Variables			Response Variables	
Run Order	*X*_1_ (%)	*X*_2_ (°C)	*X*_3_ (min)	TGC (mg SE/g DW)	Sulforaphane (mg/g DW)
**1**	79.73	67.83	51.8921	18.9	0.02
**2**	79.73	32.1619	28.1079	21.0	0.02
**3**	50	50	40	42.4	0.36
**4**	50	50	40	43.0	0.31
**5**	50	50	40	41.8	0.31
**6**	50	80	40	36.7	0.18
**7**	100	50	40	11.0	0.01
**8**	0	50	40	41.5	0.30
**9**	50	20	40	37.4	0.35
**10**	79.73	67.83	28.10	19.3	0.03
**11**	50	50	40	41.4	0.31
**12**	20.27	32.16	51.89	34.9	0.33
**13**	50	50	40	41.8	0.30
**14**	50	50	20	36.1	0.30
**15**	50	50	60	40.4	0.32
**16**	20.27	67.83	51.89	34.5	0.23
**17**	50	50	40	41.0	0.30
**18**	20.27	67.83	28.10	38.4	0.26
**19**	20.27	32.16	28.10	46.9	0.35
**20**	79.73	32.16	51.89	15.9	0.02
Predicted optimum conditions *	24.24	40.6	32.12	46.6 *	0.38 *

Note: Ethanol concentration (*X*_1_), temperature (*X*_2_), and extraction time (*X*_3_). TGC, total glucosinolate content; SE, sinigrin equivalence. * Predicted optimum conditions for maximum total glucosinolate content (TGC), which was the primary response used for optimization. The predicted sulforaphane content represents a secondary response under these TGC-optimal conditions and was not independently optimized. TGC was estimated using a spectrophotometric method [6,7]. Sulforaphane was quantified using UPLC-ESI-MS.

**Table 3:** Comparative analysis using different extraction methodologies and extraction parameters.

Extraction Method	Extraction Conditions			References
	Solvent	Temperature (°C)	Time (min)	
UAE	25% EtOH	41 °C	32	Present study *
UAE	80% MeOH	41 °C	32	[7]
UAE	70% MeOH	41 °C	32	[7]
MAE	25% EtOH	100 °C	32	For comparison with UAE
MAE	70% MeOH	100 °C	5	[8]
WBE	70% EtOH	70 °C	5	[9]
SAE	73% MeOH	25 °C	60	[10]
NADES	Choline chloride & citric acid (1:2)	70 °C	60	[11]

Abbreviations: UAE, ultrasound-assisted extraction; MAE, microwave-assisted extraction; NADES, natural deep eutectic solvent; SAE, shaker-assisted extraction; WBE, conventional extraction in a water bath; EtOH, ethanol; MeOH, methanol. Present study *—optimized conditions in the current study based on TGC assay results.

**Table 4:** ANOVA results of significance (*p*-values) of the major factors and their interactions based on the response surface methodology of the CCD model.

	*p*-Value	
Source	TGC (mg SE/g DW)	Sulforaphane (mg/g DW)
% EtOH (*X*_1_)	<0.001	<0.001
Temperature (*X*_2_)	0.525	0.057
Time (*X*_3_)	0.308	0.866
% EtOH*% EtOH	<0.001	0.001
Temperature*Temperature	0.018	0.044
Time*Time	0.037	0.256
% EtOH*Temperature (*X*_1_ *X*_2_)	0.344	0.270
% EtOH*Time (*X*_1_ *X*_3_)	0.332	0.752
Temperature*Time (*X*_2_ *X*_3_)	0.238	0.902
Lack-of-Fit	<0.001	0.010
R-Square	93.97%	89.13%
R-square (adjusted)	88.54%	79.35%

Abbreviations: TGC, total glucosinolate content; SE, sinigrin equivalence; *, interaction. TGC was estimated using a spectrophotometric method. Sulforaphane content was quantified using UPLC-ESI-MS.

**Table 5:** Comparison of the predicted and experimental TGC and sulforaphane recovery under optimized UAE conditions.

Measurement	Predicted	Actual	% Error
TGC (Spectrophotometric method)	46.6 (mg SE/g DW)	36.55 ± 4.4 (mg SE/g DW)	21.6%
Sulforaphane content (UPLC-ESI-MS method)	0.38 (mg/g DW)	0.31 ± 0.0 (mg/g DW)	18.4%

**Table 6:** Comparison of the extracts prepared using different extraction methods for total antioxidant capacity (FRAP, DPPH and ORAC).

Extraction Method	Extraction Conditions			Antioxidant Capacity		
	Solvent	Temperature (°C)	Time (min)	FRAP (µmol TE/g DW)	DPPH (IC_50_; mg DW/mL)	ORAC (µmol TE/g DW)
UAE	25% EtOH	41 °C	32	21.1 ± 2.5 ^a^	3.71 ± 0.23 ^b^	156 ± 15.2 ^a^
UAE	80% MeOH	41 °C	32	15.4 ± 1.0 ^ab^	3.11 ± 0.06 ^c^	129 ± 8.3 ^a^
UAE	70% MeOH	41 °C	32	15.9 ± 2.4 ^ab^	2.49 ± 0.22 ^de^	142 ± 8.4 ^a^
MAE	25% EtOH	100 °C	32	12.8 ± 0.06 ^b^	7.06 ± 0.16 ^a^	130 ± 77 ^a^
MAE	70% MeOH	100 °C	5	17.6 ± 3.24 ^ab^	2.39 ± 0.33 ^e^	140 ± 56 ^a^
Water-bath	70% EtOH	70 °C	5	16.2 ± 4.5 ^ab^	2.99 ± 0.09 ^cd^	142 ± 1.7 ^a^
Shaker	73% MeOH	25 °C	1 h	17.3 ± 0.69 ^ab^	2.73 ± 0.23 ^cde^	149 ± 56.3 ^a^
NADES	Choline chloride & citric acid (1:2) molar ratio	70 °C	1 h	19.0 ± 2.37 ^ab^	NA *	154 ± 95.4 ^a^

Note: Different superscript letters (a–e) within a column indicate statistically significant differences (*p* < 0.05) based on Tukey’s HSD test. Abbreviations: UAE, ultrasound-assisted extraction; MAE, microwave-assisted extraction; NADES, natural deep eutectic solvent; DW, dry weight basis; NA *, not available. For the DPPH assay, extracts were tested over a concentration range of 0.5–40 mg/mL, expressed on a dry extract mass basis, and IC_50_ values were interpolated from the resulting dose–response curves.

## Data Availability

The original contributions presented in this study are included in the article/Supplementary Materials. Further inquiries can be directed to the corresponding author.

## Supplementary Materials

The following supporting information can be downloaded at: https://www.mdpi.com/article/10.3390/foods15193431/s1, Figure S1: Normality plots.
