# Supplementary material for: Glucosinolate- and Sulforaphane-Enriched Broccoli Extract as a Potential Dual-Action Antioxidant and Elastase Inhibitor
Source: Foods. 2026 Sep 25;15(19):3431. doi: 10.3390/foods15193431 (PMC13664207; doi:10.3390/foods15193431)

Supplementary Material

Figure S1. Normality plots

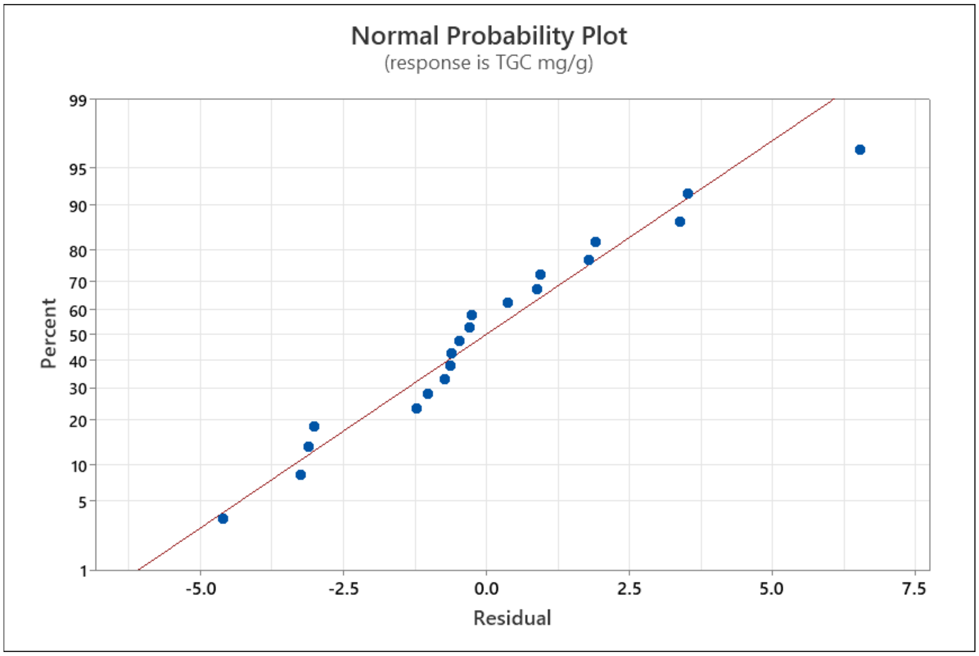

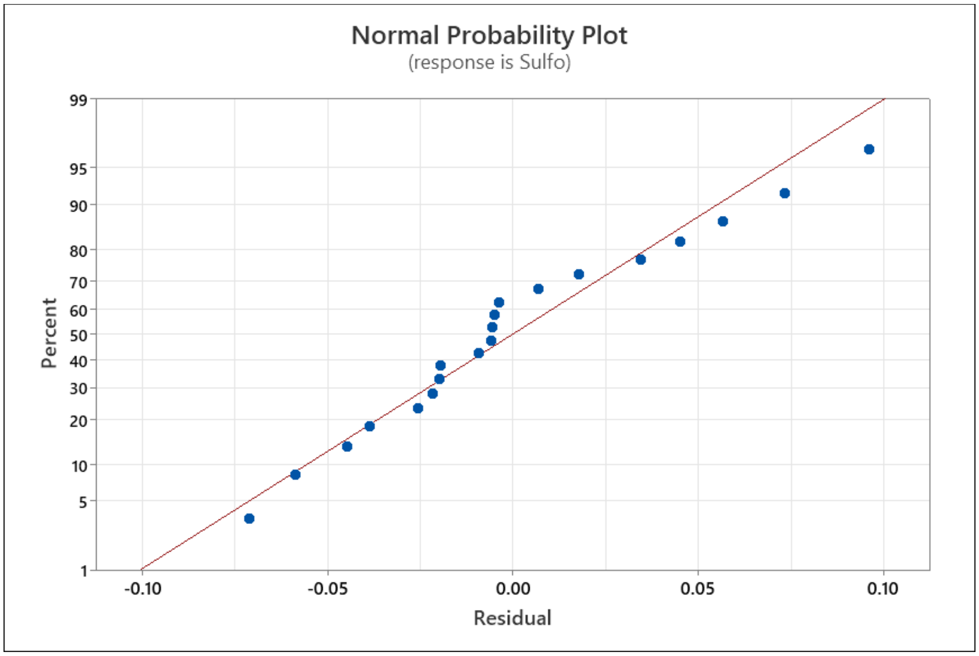

Supplement: Supplementary file 1 [file foods-15-03431-s001.zip › foods-4517597-supplementary.pdf]
